# Exploring Factors Affecting the Rollout of a Policy on Registered Advanced Nurse Practitioners in Ireland

**DOI:** 10.1155/2024/6681576

**Published:** 2024-05-28

**Authors:** Naomi Elliott, Louise Daly, Denise Bryant-Lukosius, Sandra Fleming, Jarlath Varley, Patrick Cotter, Elaine Lehane, Shauna Rogerson, David O'Reilly, Jonathan Drennan, Anne-Marie Brady

**Affiliations:** ^1^School of Nursing and Midwifery, Trinity College Dublin, The University of Dublin, 24 D'Olier Street, Dublin 2, Ireland; ^2^Ageing and Community Nursing Trinity, Centre for Practice and Healthcare Innovation, School of Nursing & Midwifery, Trinity College Dublin, The University of Dublin, 24 D'Olier Street, Dublin 2, Ireland; ^3^School of Nursing, McMaster University, Health Science Centre 2J25, 1280 Main Street West, Hamilton Ontario L8S 4K1, Canada; ^4^School of Nursing and Midwifery, Trinity College Dublin, The University of Dublin, 24 D'Olier Street, Dublin 2, Ireland; ^5^RCSI School of Nursing and Midwifery, 123 St Stephen's Green, Dublin D02 YN77, Ireland; ^6^Catherine McAuley School of Nursing and Midwifery, Brookfield Health Sciences Complex, University College Cork, Cork T12 AK54, Ireland; ^7^University College Cork, School of Nursing and Midwifery, Brookfield Health Sciences Complex, College Road Cork T12 AK54, Cork, Ireland; ^8^Faculty of Biological Sciences, School of Biomedical Sciences, University of Leeds, Leeds, UK; ^9^School of Nursing, Midwifery and Health Systems, University College Dublin, Belfield, Dublin 4, Ireland; ^10^Trinity Centre for Practice and Healthcare Innovation, School of Nursing and Midwifery, Trinity College Dublin, The University of Dublin, 24 D'Olier Street, Dublin 2, Ireland

## Abstract

**Aim:**

To identify the barriers and enablers to the implementation of a national policy to increase and develop the advanced nurse practitioner (ANP) workforce in Ireland.

**Background:**

The Department of Health (Ireland) introduced a policy to increase the number of ANPs to 2% of the nursing workforce. Evaluation provides information to inform successful policy implementation and development of ANP roles in healthcare services.

**Methods:**

Qualitative descriptive design. Twenty candidate ANPs participated in four focus groups. Nine key stakeholders were also interviewed.

**Results:**

Analysis identified four barriers: lack of infrastructural resources; delay in releasing and arranging replacements for candidate ANPs; role resistance from administration, allied healthcare professionals and other nurses; and lack of organisational readiness. The five enablers were: supportive physicians; Nursing and Midwifery Practice Development Units; supportive directors of nursing; role awareness and clarity; and educational preparation.

**Conclusions:**

This evaluation identifies barriers and enablers to the implementation of a national policy to increase the critical mass of advanced practitioners within the healthcare services. Evaluation at the implementation phase informed the roll-out of future advanced practice initiatives. *Implications for Nursing Management*. To support advanced practice development, leadership, infrastructure, and resource planning are needed to harness known enablers and address identified barriers to the implementation and sustainability of these posts.

## 1. Introduction

Recent decades have witnessed the growth of Advanced Nurse Practitioner (ANPs) roles. Globally, such roles evidence diversity in the rationale for their development, role focus and functions, role titling and terminology, education and regulation requirements [1], International Council of Nurses (ICN) 2020; [2]. This is noted in the most recent ICN [3] guidelines which aim to provide uniformity in the identification and integration of such roles within systems of healthcare. Evidence of the positive impact of the ANP role include beneficial patient outcomes, continuity and integration of care, clinical leadership and, improved efficiency and cost effectiveness [3–7]. However, to be successful, the introduction of, and succession planning relating to, ANP roles should be linked to identified service needs and involve comprehensive planning along with education and strategic and organisational supports, and evaluation [8, 9]. An increasing empirical focus on the factors that can support the implementation and development of the ANP role is evident. This paper reports on the qualitative findings from an evaluation of the introduction of a policy to increase the number of ANPs in key patient areas [10]. Specifically, the research identified barriers and enablers to the development of the role from the perspective of candidate ANPs (cANP), registered ANPs and key stakeholders.

## 2. Background and Context

In the Republic of Ireland, the registered ANP role was first accredited in 2002 and is defined as a “career pathway for registered nurses, committed to continuing professional development and clinical supervision, to practice at a higher level of capability as independent, autonomous, and expert practitioners” Nursing and Midwifery Board of Ireland (NMBI). Registered ANPs have competencies that enable them to be senior decision makers, work collaboratively, demonstrate clinical leadership and research skills, undertake advanced physical and/or mental health assessments, and implement evidence-based interventions including prescribing when needed with service users that have complex and multiple needs [11]. Those holding the role are regulated and subject to the Advanced Practice (Nursing) Standards and Requirements published by the NMBI [12] for education and registration of ANPs. To meet the increasing demands within the health service and acknowledging the impact that ANPs were having on patient care, the Department of Health published the document Developing a Policy for Graduate Specialist and Advanced Nursing & Midwifery Practice: Consultation Paper [13]. This consultation paper outlined a new structured model for graduate to advanced practice to support the development of a critical mass of nurses and midwives to “address emerging and future service needs and drive integration between services” (p.8). This document identified a goal of increasing the number of ANPs to 2% (*n* = 700 approximately) of the nursing and midwifery workforce by 2021 and led to the roll-out of a two-year demonstrator project (the subject of this evaluation). This was followed by the publication of A Policy on the Development of Graduate to Advanced Nursing and Midwifery Practice in 2019 (Department of Health). In Ireland, those nurses preparing to be ANPs are titled “candidate ANPs'; this refers to a nurse who is undertaking an advanced nursing practice Master's degree education programme which includes both academic and advanced clinical competency requirements. Candidate ANPs are employees within the health services and to undertake the education programme, need protected study leave and to be released from clinical practice.

Evidence suggests that considerations pertinent to the support of ANP roles are both complex and multiple. A scoping review by Whitehead et al. [14] focusing on factors that provide a positive impact identified the importance of a robust candidacy programme, clarity of roles which are identified to address a clear gap in the provision of healthcare, interdisciplinary integration, strong mentorship and ongoing professional development. In Australia, a national system for regulating the credentialing of Nurse Practitioners which sets the standards for assessment and competency requirements is identified as an important factor in supporting advanced practitioner role development [15]. Similarly, Lee et al. [16] comparative analysis of nursing regulatory frameworks between Mexico and the United Kingdom highlights the importance of having regulatory frameworks to ensure a coherent and uniformly understood approach, thereby optimising the development of advanced practice nursing that meets health care needs. Findings of a study of role satisfaction, highlighted that empowerment-related factors were also critical, including collegiality, professional growth and research [17]. Other studies have identified the importance of organisational readiness and policies, governance and future planning in relation to succession and regulation [1, 9, 18, 19]. An understanding of such factors is pivotal to the success of the ANP role and the redress of healthcare needs if sustainable change is to be achieved [14, 20]. Therefore, evaluation of innovations to grow ANP roles and related experiences are imperative to understand factors that enable the development, implementation and sustainability of the role within local contexts. This paper reports on the findings from one part of a multicomponent evaluation study [10] that examined the impact of the national consultation document and the development of a policy to develop ANPs in four key areas: older person's care, unscheduled care, and respiratory and rheumatology [13]. The aim of the component reported herein was to explore ANPs' and other stakeholders' perspectives on the implementation of the policy. The aim was achieved by applying a qualitative descriptive methodology.

## 3. Methods

### 3.1. Design, Setting and Participants

A qualitative descriptive approach was used as it enables the exploration of research participants' perspectives on issues of importance aligned to research questions [21]. The main evaluation study design [10] was informed by the Participatory Evidence-Informed Patient-Centred Process for Advanced Practice Nursing Role Development (PEPPA) and PEPPA-Plus frameworks which were designed to inform the development, implementation, and evaluation of ANP roles [8, 22]. The PEPPA framework emphasizes strategies to mitigate frequently reported barriers to effective ANP role design and implementation, while PEPPA-Plus provides enhanced guidance for role evaluation. The PEPPA framework has been used in over 25 countries to introduce a variety of advanced nursing and other healthcare provider roles with reported benefits of framework related to role clarity, stakeholder role acceptance and support, and successful role implementation [23]. A freely accessible online toolkit [24] provides detailed guidance on how to implement the stepwise, systematic framework. The frameworks incorporate Donabedian's [25] theory and provide conceptual clarity by distinguishing the structure-process-outcome aspects of the ANP role development that have important considerations within the evaluation process. Importantly, the frameworks provide clarity by distinguishing between short-term, intermediate-term and long-term outcomes of the ANP role which inform the goal and direction of the evaluation.

At policy level, PEPPA provides an evaluation framework that can be used at the three stages of role development namely, (1) introduction, (2) implementation and (3) sustainability. Use of the framework was therefore appropriate to the aim of the overall study, while providing a baseline dataset against which future evaluations can be used to compare progress on factors that influence key outcomes such as waiting list reduction, timely access to services and avoidance of unnecessary hospital admissions.

### 3.2. Data Collection

In the qualitative component, semi-structured focus group and individual in-depth interviews were conducted to gather data pertinent to experiences of the implementation process, factors impacting upon it and outcomes. Participants were recruited via purposive and snowball sampling methods. Interview guides were developed by the research team from the study aim and objectives, and previous literature on ANPs (see Table 1). The use of interview guides provided for consistency across interviews.

Participants from each of the four key areas (older person's care, unscheduled care, respiratory, and rheumatology) were sought via local gatekeepers. Area managers within Nursing Practice Development where candidate ANPs were recruited, acted as gatekeepers. The sampling strategy used was designed to provide for a national spread, geographical and clinical area diversity and the concept of information power was used to determine data sufficiency [26]. Candidate ANPs were employed in acute healthcare settings, out-patient services, and a number were developing links with community care services. Focus groups were held with each of the four areas in which ANPs were recruited as consequence of policy implementation: older person's care (*n* = 5), respiratory (*n* = 5), rheumatology (*n* = 6) and unscheduled care (*n* = 4). The four focus groups were each facilitated by two research team members and were held face-to-face. Individual face-to-face or telephone interviews (*n* = 9) were held with other key stakeholders. The key stakeholders provided perspectives of those with first-hand experience of the ANP policy implementation at individual ANP, local and national levels including clinician candidate ANP supervisors, medical consultants, allied healthcare professionals, and senior nursing managers in local and national settings.

### 3.3. Data Analysis

Template analysis was employed to explore the data with reference to the study aim. This form of thematic analysis focuses on research conducted within real-world contexts and facilitates the definition and organisation of themes [27, 28]. It is located midway on the inductive-deductive continuum. The approach enables structure and flexibility in how data can be analysed. Template analysis is operationalised via seven steps that were applied as outlined in Table 2 by two of the research team with extensive experience in qualitative research. NVivo 12 Plus [30] software was employed to facilitate the analytical interrogation and management of data.

### 3.4. Ethical Considerations

Ethical permission to conduct the study was obtained from a university ethics committee and relevant local research ethics committees. Participants were provided with Participant Information Leaflets, with explicit and process approaches to informed consent applied, along with the principles of voluntariness and confidentiality in relation to study participation, data processing and study write-up.

### 3.5. Findings

Data analysis identified the key barriers and enablers to the implementation of the ANP draft policy as listed in Table 3.

### 3.6. Barriers to ANP Draft Policy Implementation

The analysis identified four key barriers to the implementation of the policy: lack of infrastructure resources; delay in releasing and arranging staffing replacement for candidate ANPs; role resistance from administration, allied healthcare professionals and other nurses; and lack of organisational readiness.

Candidate ANPs and key stakeholders identified a lack of infrastructure resources such as clinic space, administrative support, office space and a specific ANP administrative identifier code as major barriers to the candidate ANP's ability to develop ANP-led clinics and manage patient caseloads. The lack of clinic space, which is essential for clinical assessments and patient treatments, and administrative support for ANP-led clinics to schedule patient appointments, manage check-in services, patient charts or type referral/letters which were available to medical teams, was perceived as limiting the candidate ANP's ability to effectively fulfil their role.

*Organizationally, the resources and the space for people has been a really sticky one for some of the candidates not being able to [have] protected space for assessment and management of patients.* (Key Stakeholder 4)

Delays in release and staffing replacement arrangements for candidate ANPs to undertake their education programme and engage in role development were also identified as hindering the process of ANP policy implementation. Because the launch of the policy was rapid, many replacement arrangements were not in place and candidate ANPs frequently found themselves needing to continue working in their previous clinical nurse specialist (CNS) or staff nurse role whilst at the same time, undertake the ANP education programme and start-up new ANP-led patient services. These delays not only created role confusion with physicians, nursing colleagues and patients, but also reduced the time available for candidate ANPs to set-up new clinical services. At local level, delays with staffing replacement arrangements were linked to the need to fill the vacant CNS posts and overall challenges with succession management.

*It took almost a year* for *us to actually start [the candidacy] in the knowledge and experience appropriate for the ANP role.* (ANP Focus Group 1)

At local level, some candidate ANPs reported having experienced role resistance from other grades such as administration, allied healthcare professionals and other nurses. In healthcare organisations with little or no previous experience of ANPs, participants reported that administrative services were frequently not available to support ANP-led clinics; this was reported to result in a reduction in the number of patients treated by ANPs. Where there was role resistance from nursing colleagues, resistance in recognising the ANP's extended role including authority for patient treatments was also reported. In some cases, allied healthcare professionals were identified as reluctant to accept patient referrals from ANPs, requests for laboratory tests, X-ray ordering or medication prescriptions.

*Radiology was a huge area… ordering a chest X-ray…I would have been probably the first one to go through in my hospital. I'm almost two years into the job and literally got it this month, approval to order x-rays.* (ANP Focus Group 2)

Due to the accelerated start-up of new candidate ANP roles, organisational readiness was reduced, and many did not have the optimal lead in time to establish governance structures to support role implementation. This also impacted on an organisation's readiness to manage problems that could arise, for example, with clinical supervision or delays in candidate ANP's progression to registration. Whilst formal service-level agreements between the director of nursing, candidate ANP and clinical supervisor were a pre-requisite in the application process for new ANP posts, the overall governance structures were, in places, under-developed at the time of the new policy implementation. Participants reported that having clear governance structures are important to provide clear pathways of communication to help resolve problems. This was also identified as important to provide clarity within the local organisation about the candidate ANP's extended scope of practice such as ordering x-rays and computerised tomography scans, prescribing medication and making patient referrals to other healthcare professionals.

*I don't think the hospital was prepared for the policy…. we came in on the basis of the policy, but the hospital wasn't prepared for that and hadn't the mechanisms for the structure set up to support it.* (ANP Focus Group 4)

### 3.7. Enablers to ANP Policy Implementation

The analysis identified five key enablers to the implementation of the policy: supportive physicians; the Nursing and Midwifery Practice Development Unit (NMPDU); supportive directors of nursing; role awareness and clarity; and the ANP preparation educational programme.

Physicians, who acted as a clinical supervisor and mentor for candidate ANPs, were identified as one of the factors that were essential to the successful implementation of the policy. Physicians provided support throughout role development and integration by identifying new service needs, negotiating with senior management for resources for candidate ANPs, encouraging other physicians to support ANP initiatives and organising clinical rotations for the education programme. At an individual level, physicians also provided personal encouragement and support. Physicians who participated in the interview and had trained in other countries with established advanced nursing practice, had a clear understanding of what the ANP role involved, how it could benefit patient services and were active champions of the new cANP roles locally.

The NMPDU and the Unit's project officers were also identified as instrumental to successful policy implementation. These units are part of the Health Services Executive working under the Office of the Nursing and Midwifery Services Director and have a remit to provide strategic development of the nursing and midwifery workforce through workforce planning initiatives and building leadership capacity within nursing and midwifery in Ireland. They supported the candidate ANPs as they transitioned from the role as clinical nurse specialist (CNS)/staff nurse to Registered ANP by guiding them through the application process, clinical portfolio requirements, how to navigate local settings and set agendas for meetings with senior management. At an organisational level, the NMPDU provided a range of supports including templates for job descriptions, memoranda of understanding for clinical supervision so that directors of nursing and candidate ANPs had clarity regarding the role and could use these standard templates to save time from having to design new documents. The NMPDU also supported the development of national standards for competencies required at ANP level which provide role clarity for all stakeholders including physicians supervising candidate ANPs and directors of nursing for signing-off candidate ANPs as being ready for registration.

*We have nationally an advanced practice network within the Office of Nursing and Midwifery Service Directorate…. When the policy was launched, and the demonstrator site project was launched, we would have supported directors of nursing in their business cases for applications for candidates.* (Key Stakeholder 5)

Directors of nursing who facilitated the new ANP roles were also identified as important enablers to the successful implementation of the ANP policy. In hospitals where directors actively supported the new roles, they did so by managing the prompt release of candidates to undertake the programme, facilitating candidate ANPs to navigate barriers, maintaining interest in their progress and providing clear leadership in multi-disciplinary meetings to ensure the effective integration of new ANPs into the healthcare organisation.

*Support* from *their organizations is essential and that's why you need your directors of nursing tuned in very much into what the role is and they [ANPs] need to be part of the senior nursing team.* (Key Stakeholder 2)

Role awareness and clarity on how the ANP role differed from other nursing roles was important to the successful integration of new candidate ANPs into the organisation. In organisations with established ANP posts, role awareness at senior nursing management and physician levels was clear and barriers for new candidate ANPs were reduced. For candidate ANPs working in organisations with little or no experience of the role, they actively worked at raising awareness about their role and managing expectations through various strategies including developing referral pathways to differentiate ANP and CNS roles, presenting at interdisciplinary meetings and conferences and developing job descriptions with consultant involvement.

The education programme, which was set by the national standards and requirements for the ANP [12], was also identified as a key enabler. It was perceived by participants as providing clarity on the academic and clinical requirements of the role, and the competencies for advanced clinical skills and caseload management.

*The* training *was fantastic. I just love what it's opened up*. (ANP Focus Group 4)

## 4. Discussion

Based on the PEPPA and PEPPA-Plus frameworks [8, 23], this study [10] was carried out at the implementation phase of the new policy to increase the number of ANPs in the health service in Ireland [31]. This paper examined the barriers and enablers to policy implementation identified from the evaluation to better understand the factors influencing the development and integration of new ANP roles into the health services. As in the 2022 report of the Expert Review Body on Nursing and Midwifery [32], the Minister for Health recommended a further increase in the numbers of ANPs, from 2% to a revised target of 3% of the nursing workforce. This highlights the importance and relevance of findings of the evaluation study to support the further development of the ANP role in Ireland. The evaluation findings provide evidence which could inform future roll-out of such policy programmes in Ireland and may also have implications for similar programmes internationally.

A key feature of the ANP policy in Ireland [31], was the role of national organisations, in particular NMPDUs in leading the process of policy implementation. Although ANPs had been employed in Ireland since 2002, for several years the posts were limited. Following the publication of the policy and the increase in posts, this resulted in many of these new ANP roles being introduced into local healthcare organisations not accustomed to starting-up such posts and to clinical areas with few or no existing ANPs to provide mentoring to the candidate ANPs. Thus, the NMPDU guidelines and templates, including ANP job descriptions, provided organisational-level support to directors of nursing who had to set up these new roles rapidly in their organisations and avoid time delays with having to develop job descriptions from scratch. The NMPDU guidelines and templates also provided for consistency across the introduction of the new ANP roles across different clinical areas and multiple healthcare organisations. At candidate ANP-level, the Project Officers within the NMPDU were key in providing mentorship and supporting candidate ANPs as they transitioned from roles as clinical nurse specialists/staff nurses to that of ANP roles involving patient caseload management, and clinical and professional leadership. Within the international literature on advanced practice, support from mentors or preceptors is recognised as a key factor influencing the successful development and integration of these roles [14].

The role of physicians who provided clinical supervision and mentoring was a key factor enabling policy implementation. However, as the numbers of ANPs increase it is expected that ANPs themselves will become clinical supervisors for future candidate ANPs. Recognising the need for having a coherent approach to clinical supervision, Lee et al. [33] highlight the importance of having national standards for supervision session structures and documentation tools thereby ensuring consistency across different healthcare organisations and universities. Clinical supervision is one factor that may ultimately affect the long-term sustainability of ANP role development and future policy implementation. The barriers to sustainability and progression of advanced nurse practitioners are complex and multi-factorial and include lack of funding and lack of medical, nursing, and organisational support [34]. The issue of clinical supervision and ensuring quality clinical supervision needs to be central in the design and delivery of ANP education programmes to ensure the sustainability of these roles.

With the introduction of new ANPs, major governance and process changes were required so that they could fulfil their extended scope of practice that included prescribing medications, ordering diagnostic tests and X-Rays, and making patient referrals to other healthcare professionals. This impacted on other healthcare professions and structures such as the Local Implementation Groups (LIGs) which were established to provide a multidisciplinary forum where issues could be discussed with the key stakeholders and problems clarified, explored, and resolved with locally mediated solutions. Similarly, Smith et al. [35] in a study of rural nurse practitioners in Australia, identified local health service managers and community supports as important enablers which can define the parameters of advanced nurse practitioner service delivery. In the Irish context, the LIG had the potential to maximise ANP role clarity and understanding within the local healthcare organisation and minimise the likelihood of role resistance.

Candidate ANPs in this evaluation experienced a number of barriers to their role. This may have been due, partly, to the relatively quick implementation of the policy and time needed by the health service to develop organisational readiness. There is growing evidence of the importance of organisational support and change management processes facilitated by leadership to the successful integration of advanced practitioners [14, 36–38]. Similarly, in this study, senior management were identified as having a key role in fostering organisational support and dealing with issues such as lack of recognition or role resistance which can have a negative impact on ability of ANPs to fulfil their role and workload expectations.

The policy initiative to increase the number of ANPs has the potential to greatly enhance healthcare service delivery in Ireland in the long-term. However, for the implementation of such initiatives to be successful, this will require continued support at national, local and individual levels [35, 37]. In Ireland, this initiative has benefitted from having a clear national policy and regulatory systems, targeted clinical areas, and funding for the new ANP roles in place at the outset. It also benefitted from having the necessary numbers of interested and eligible candidates who were motivated to advance their clinical careers. Although this evaluation study was carried out in the early implementation stage, some candidate ANPs were beginning to express concerns arising from the need for enhanced organisational supports, which, if not in place, could negatively impact on the long-term sustainability of the national policy programme to increase the number of ANPs in the nursing workforce. Whilst the factors influencing job satisfaction are complex, a national survey of advanced nurse practitioners in Taiwan found that organisational commitment was ranked as the most important work-related variable linked to job satisfaction [39]. Similarly, Wood et al. [40] study of the experiences of UK nurse practitioners, links support for advanced practitioners with levels of satisfaction with the role. Creating an organisational culture that is open to the integration of new ANP roles is an ongoing process that will take time. It is recognised that this evaluation was carried out in the early stages of a rapid policy implementation, however, it is also recognised that the long-term sustainability of the policy to develop a critical mass of ANPs to address emerging and future health service needs is at risk unless ANPs are supported adequately by senior management and the healthcare organisation.

## 5. Conclusion

This evaluation presents findings on the key barriers and enablers to the implementation of a policy to increase the number of ANPs in Ireland. Implementation of national policy initiatives within healthcare organisations is challenging and complex. Policymakers, healthcare funders and organisations need to evaluate such initiatives so that lessons learnt can inform and improve the future development and integration of ANPs within the healthcare system. The knowledge generated contains transformative potential in that it can inform the future design and support of such roles so that they meet evolving healthcare needs and enable ANPs to function independently within their scope of practice.

## Figures and Tables

**Table 1:** Semi-structured interview question guide.

Q1	What motivated you to pursue this cANP/ANP role?
Q2	Can you explain how you were recruited?
Q3	Tell us about what is different about your cANP/ANP role in the context of this service?
Q4	Have you experienced any challenges in your new role?
	What strategies did you use to overcome those challenges?
Q5	What organisational factors enabled you in your role to date?
Q6	What are the arrangements for supervision, support and mentorship with your new role?
Q7	Can you comment on the specific contribution of your role to your specialty area?
Q8	What do you see as the specific contribution since the cANP/ANP role was implemented to: patients/families; the multidisciplinary team; the health service organisation?
Q9	Can you describe the impact of your cANP/ANP role on health service needs?
Q10	If the policy was to be expanded what one thing would you recommend to enhance the process?

**Table 2:** Template analysis [28].

Template analysis step	Application in the current study
1. Data familiarisation and identification Identification of *a priori* themes	Two of the research team listened to, read and re-read the interview transcripts to facilitate early engagement with and reflection on the interview and focus group data The taxonomy of implementation outcomes [29] and advanced nursing practice literature informed the identification of soft *a priori* themes (potentially relevant aspects of the data)

2. Preliminary coding	Recurring concepts in the data relating to the research purpose (within and between transcripts) were identified, discussed and operationally defined in NVivo. Codes from the tentative *a priori* themes were tested against the data

3. Clustering	Developing themes were organised into meaningful clusters. Researchers explored how themes might relate to each other

4. Production of the initial template	As clustering advanced, researchers reached consensus on the initial template

5. Developing the initial template	Openness was maintained to facilitate ongoing refinement of the template

6. Applying the template	Organisation of hierarchical themes advanced in tandem with revisiting the interview transcripts to ensure thorough application of the template

7. Write up	The analytical interpretation was written up, discussed with reference to the study aim and objectives by the overall research team and finalised

**Table 3:** Key Barriers and Enablers to ANP draft policy implementation.

Key barriers	Key enablers
(i) Lack of infrastructure resources; (ii) Delay in releasing and arranging staffing replacement for candidate ANPs; (iii) Role resistance from administration, allied healthcare professionals and other nurses; (iv) Lack of organisational readiness	(i) Supportive physicians; (ii) Nursing and midwifery practice development unit (NMPDU); (iii) Supportive directors of nursing; (iv) Role awareness and role clarity; (v) ANP preparation educational programme

## Data Availability

The qualitative data used to support the findings of this study are included within the article.

## Additional Points

*Implications for Nursing Management*. To support advanced nurse practitioner development, leadership, infrastructure and resource planning are needed to harness known enablers and address identified barriers to the implementation and sustenance of related posts. Lessons learnt from evaluation at the implementation phase of new policies can inform the future roll-out and long-term sustainability of developments to build advanced nurse practitioner capacity and realise the potential of nurse practitioners in delivery of healthcare.
